# Factors that relate to sport participation of adolescents with a mobility impairment

**DOI:** 10.4102/ajod.v8i0.614

**Published:** 2019-09-23

**Authors:** Aletta M. Moll, Garfield Bester

**Affiliations:** 1 Department of Psychology of Education, College of Education, University of South Africa, Pretoria, South Africa

**Keywords:** mobility impairment, sports participation, adolescents, adaptive sport, disability

## Abstract

**Background:**

There are multiple factors that make it difficult for learners with a mobility impairment to participate in sport, if not impossible. Unfortunately, it is not known which of these factors can be considered as the most important ones.

**Objectives:**

The main objective was to obtain clarity on the factors that differentiate best between learners who participate in sport and those learners who are not participating.

**Method:**

In total, 140 boys and girls with different types of mobility impairments participated. Information was obtained on *inevitable factors* such as age and gender, *structure factors* such as type of school and hostel dwelling and personal factors such as emotions and relationships with parents and peers.

**Results:**

Four factors emerged that explained 22% of the variance in the distinctive characteristics of the group that participates in sport and the non-participating group. Age was the most important variable explaining 9% of the variance followed by trust (an emotional variable), gender and health.

**Conclusion:**

Children with a mobility impairment should be encouraged to start participating in sport at an early age. Specific attention should be given to girls who are more reluctant to participate. Health is a factor that can inhibit sports participation; however, it should not be overemphasised. The emphasis should rather be on the development of trust, which will help adolescents with an impairment to take responsible risks in an adaptive sports environment.

## Introduction and background

The development of children incorporates a physical component that requires schools to involve children in a variety of physical activities, exercise programmes or sport participation initiatives. Zourikian, Jarock and Mulder (2012:12-1) differentiate between physical activity, exercise and sport in the following manner: Physical activity is defined as any activity that comprises some form of physical effort and voluntary movements that contribute to energy expenditure, for example walking, dancing or any activity causing a person’s body to work harder than normal. Exercise also involves physical action, voluntary movements and energy expenditure. This form of physical activity is specifically planned, structured and repetitive. Generally, it does not involve any kind of competition. According to Zourikian et al. (2012:12-1) sport also involves physical activity and exercise but in sport, there are specific rules that should be adhered to and training programmes that should be followed to excel and reach particular goals (Zourikian et al. 2012:12-1).

Participation in physical activity, exercise or sport has numerous benefits for individuals. The most positive outcomes are the improvement in physical health, strength building, enhanced coordination and motor skills, and improved cardiovascular health (Allender, Cowburn & Foster 2006:826). Virgilio (2012:5) identified the following physical benefits from being physically active: weight control, controlled blood pressure, reduced risk of heart diseases, avoidance of some cancers and type 2-diabetes, reduced cholesterol levels and the development of strong bones and muscles. Another important aspect of sport participation or being involved in any kind of physical activity is the impact that it has on emotional healing and psychological wellbeing (Coakley & Dunning 2000:477). Sports participation provides a positive outlet for aggression and stress and helps alleviate depression and anxiety. Not only does it improve mental functioning and concentration but also facilitate self-confidence and a positive self-image (Coakley & Dunning 2000:477). Athletes with a disability who participate in sport exhibit higher levels of positive mood, increased wheelchair mobility skills, show lower levels of tension and depression, and an improved state of health and wellbeing (Groff, Lundberg & Zabriskie 2009:320, 324‒325).

Participation in sport also plays a significant role in healthy social development and interaction. Allender et al. (2006:826) found that although most people recognised the health benefits, this was not their main reason for participation in sport. The factors such as enjoyment, social interaction and support were more common reasons for participation in sport.

In light of the importance and benefits of sport, it is alarming to see that children in general are becoming less involved in sport. According to McVeigh and Norris (2012:43), South African children show trends of obesity and overweight and less than one-third of the children participate in sufficient physical activity on a weekly basis. Draper et al. (2014) also reported on the decline of physical activity and concluded as follows:

South Africa has moved from a C [grade] in 2010 to a D grade in terms of getting children physically active and eating healthily. The time has come for engaging parents and communities for advocacy and social mobilization. (p. S104)

Previous research indicates various reasons for non-participation in sport. Crawford and Godbey (1987:8) identified three categories of constraints: *intrapersonal* (a lack of self-confidence, encouragement or a lack of information about opportunities), *interpersonal* (lack of leisure partners or social interaction skills) and *structural* (lack of finances, transportation or lack of time). Kirk and Kirk (1993:86) established that certain internal factors such as low self-esteem, lack of confidence, lack of general information, conflict between personal values and athletic goals, fear of failure and lack of decision-making skills could be regarded as possible barriers. Singer, Hausenblas and Janelle (2001:517) identified the following possible barriers with regard to sport participation: lack of physical skills, lack of confidence, unrealistically difficult goals and too many vague and conflicting goals. A number of external factors can also be identified as possible barriers which prevent sport participation. Such factors are lack of role models and mentors, stereotyping, discrimination, admission criteria, socio-economic status, family expectations and peer pressure (Kirk & Kirk 1993:86). Singer et al. (2001:517) identified barriers such as lack of time for proper training, personal and family responsibilities and lack of social support.

The barriers mentioned may or may not apply to athletes with a mobility impairment. Mobility impairment is a category of disability that includes people with varying types of physical disabilities. It specifically refers to the inability of a person to use one or more of his or her extremities, or a lack of strength to walk, grasp or lift objects (Colorado State University 2016:1). Mobility impairment can include an inability to move around as easily as others, limited movement of arms or legs, decrease in strength or control of the muscles and abnormal or impaired coordination. It is, thus, a disability that interferes with a person’s ability to perform tasks that require motor control and coordination. In some adolescents, the impairments are visible and evident and in other adolescents, the impairment may be less obvious. The use of mobility aids such as canes, crutches, walkers, wheelchairs and scooters is normally an indication of the severity of the disability (Davis et al. 2011:80).

To facilitate sport participation among adolescents with a mobility impairment, barriers should be eliminated as far as possible. Moran and Block (2010:2) mention four of the most common barriers. Firstly, the coaches fear liability. They fear a player with a disability will get hurt, which may result in parents taking legal action or that the athlete’s adaptive equipment (crutch or artificial arm) would injure another athlete. Secondly, many coaches agree that athletes with a disability deserve ‘the right to participate’ but they lack the knowledge to appropriately meet their needs. Thirdly, parents are concerned regarding the remarks of other athletes about their own child’s disability. Parents also fear for their child’s safety as they are concerned that their child might get hurt or harm other children. Fourthly, there is a lack of appropriate programmes, especially in small rural areas.

Each form of disability and mobility impairment has different requirements, which complicate matters even more. Skučas (2013:85) identified the reasons that spinal cord injured athletes gave for not participating in sport. The reasons were a lack of adapted sports facilities, limited independence, lack of time, financial problems, transport problems and lack of coaches and sports specialists. In a study conducted by Stephens, Neil and Smith (2012:2067) among permanent wheelchair users, the following barriers to sport participation were identified; medical barriers, emotional barriers, a lack of information and stereotype views held by others.

Mainstreaming was one of the early models used to accommodate learners with a disability in the regular educational environment. Mainstreaming is defined as the integration of learners with a disability into the ordinary school without changes in curriculum, organisation or teaching strategies (Department of Basic Education 2011:51). Mainstreaming, as well as the later approaches of integration, stems from the normalisation principle, which states that people with a disability have the right to life experiences that are the same as, or very similar to, those of others in society (Landsberg, Krüger & Nel 2005:7). The goal with mainstreaming in an educational environment is to include learners with a disability in the mainstream as far as possible alongside normal developing peers. This also applies to a sports context. The implication of mainstreaming, according to Pangrazi and Beighle (2014:13), is that learners with a disability can participate in physical education and sporting events on an equal level with peers not having a disability, without any changes or adaption of the activities. However, it is not always possible to accommodate all learners successfully without any adaption. It is thus understandable that mainstreaming has been criticised for neglecting to provide for learners with a disability.

## Aim of the investigation

From the available literature, it seems that there are multiple factors that make it difficult, if not impossible for learners with a mobility impairment to participate in sports. Unfortunately, it is not known which of these factors can be considered as the most important ones. To determine the importance of these factors it would be helpful to categorise the factors in some way or another. In the current investigation, the possibility of three categories is proposed. Firstly, there are *inevitable* factors that cannot be changed, treated or manipulated such as age, gender, type of mobility impairment and the onset of the disability. Secondly, there are factors related to *structure* (Crawford & Godbey 1987:8) such as the type of school, hostel dwelling, transportation, finance and the availability of mentors or coaches. Thirdly, there are *personal* factors (Crawford & Godbey 1987:8) such as emotional stability, self-confidence, goal orientation, self-concept, fear of failure, depression, relationships with parents and relationships with friends. If the factors are categorised, the question arises as to the importance of these categories or the factors in each of the categories. The current investigation was, therefore, planned to obtain clarity on the factors that differentiate best between learners who are willing to participate in sport and those learners who are not interested. With this intention in mind, the first aim of the investigation was to establish how certain factors in each category facilitate or inhibit sports participation of adolescents with a mobility impairment. The second aim was to identify the most important factors in this regard.

## Research method

### Sampling procedure and ethical considerations

Certain conditions were set regarding the selection of the respondents. The respondents had to be adolescent boys and girls. There were two reasons for focusing on adolescents. Firstly, sports involvement becomes more constructive and organised during adolescence, which compels adolescents to make a choice regarding their participation. Secondly, some of the measuring instruments (such as the Emotional Profile Index) need objective introspection that might be problematic for younger learners to answer. Another condition was that the respondents had to be proficient in either Afrikaans or English. The questionnaires used to gather the data were only available in the two languages since most learners in South Africa are taught in either of the two languages. Lastly, the respondents had to have a mobility impairment. It was decided that any form of mobility impairment could be included in the sample, ranging from the most severe disability to an almost unobtrusive impairment. Learners who were mobility impaired but also suffered severe intellectual limitations, which would have made it impossible for them to understand the items in the questionnaire, were excluded from the sample.

Due to the conditions mentioned above, purposeful sampling had to be used. Some of the schools that accommodate adolescents with a mobility impairment also accommodate adolescents who do not have a mobility impairment, which makes random sampling impossible. Other schools accommodate adolescents with a mobility impairment and adolescents with intellectual challenges which again makes it difficult to conduct random sampling.

Schools that accommodate learners with special educational needs in the different provinces of South Africa were included in the sample. These schools were identified from the databases provided by the respective Provincial Departments of Education. Once possible schools were identified, the respective Departments of Education were approached to obtain permission to conduct the research in their districts. Most of the departments granted permission for the research. The school principals were then approached. Once the principals granted permission, the parents were approached to seek their permission and finally the willingness of the learners to participate in the research was ensured. Consent was obtained from all the participants who took part in the investigation. Confidentiality was guaranteed and no identifiable information was made available. The only risk for the participants was the inconvenience for some of them to complete the questionnaire. Every participant had the right to withdraw from the research at any time. Ethical clearance was granted by the university under whose auspices the study was carried out.

An attempt was made to include all the adolescents with a mobility impairment in each of the selected schools. A further attempt was made to assist selected learners to complete the questionnaires in order to keep the attrition of participants to the absolute minimum. In total, 140 (78 boys and 62 girls) learners participated. Their age ranged from 14 to 20 with an average age of 16.81 and a standard deviation of 2.15. The provinces, the number of schools and the number of learners included in the final sample were: Gauteng (8 schools, 74 learners), North West (1 school, 13 learners), Northern Cape (1 school, 11 learners), Eastern Cape (1 school, 14 learners) and Free State (1 school, 28 learners).

Different types of mobility impairment were included in the sample. Cerebral palsy, caused by abnormal development of the brain, was the most common mobility impairment among the respondents in the sample. The number of respondents in each category of mobility impairment was: Hemiplegia (15), Paraplegia (15), Quadriplegia (6), Cerebral palsy (44), Spina bifida (and other bone deformities) (25), Muscular dystrophy (and other muscle weaknesses) (22), Amputee (5) and Multiple sclerosis (8). The majority of the respondents (72%) had a congenital impairment compared to 28% who acquired the mobility impairment after they were born.

### Measuring instruments

The questionnaires used in the investigation correspond with the three categories previously identified. To measure *inevitable* factors respondents had to provide general information about themselves such as gender, age, school grade and home language. They were asked to indicate which mobility impairment they had and whether it was a congenital or an acquired impairment. Respondents also had to rate their current state of health on a six-point scale ranging from good to bad.

The respondents had to indicate whether they currently participate in sport. The term sport participation was explained to the learners and typical examples of sport offered by most of the schools were listed in the questionnaire. The learners also had the opportunity to add other sport types they engage in to the list. After the information on their sport participation was obtained, information on *structure* factors was required such as the environment in which they participate (at school or at a venue away from school; with other learners who have a disability and/or learners without a disability). Information on the type of school (mainstream or special needs school) and hostel lodging were also obtained.

To obtain information on *personal* factors, the emotional profile for each respondent was obtained. A questionnaire to measure the relationships with parents and peers was also conducted.

#### Emotional profile

The Emotions Profile Index (EPI) is a measuring instrument developed by Plutchik and Kellerman (1974). They identified eight primary emotions which were coordinated in pairs of opposites. The index consists of 12 traits, which are paired in all possible permutations, through a 62-item forced-choice questionnaire. The 12 traits are adventurous, affectionate, brooding, cautious, gloomy, impulsive, obedient, quarrelsome, resentful, self-conscious, shy and sociable (Kellerman & Plutchik 1968:1109–1110). A definition for each trait is provided. The respondent is asked to indicate his or her preference for one of two traits. Each time a trait is chosen, the score on one or more of the eight basic emotional dimensions increases (Kline 2000:339). From the 12 traits, 8 basic bipolar emotional dimensions are derived (Louw 2004:69). They are:

- timid (protection) versus aggression (destruction)
- trustful (incorporation) versus distrust (rejection)
- control (exploration) versus discontrol (orientation)
- gregarious (reproduction) versus depression (reintegration).

The test-retest reliability of the EPI provided a reliability coefficient 0.9 and the split-half reliabilities for the different dimensions were timid (0.80); aggression (0.77); trust (0.89); distrust (0.61); control (0.78); discontrol (0.75); gregarious (0.90) and depression (0.71) (Louw 2004:75, 2015:34).

#### Relationships with parents and peers

Fourie (2001:178) compiled a parent-adolescent relationship questionnaire to obtain information from adolescents regarding their relationships with their parents. The questionnaire consists of 43 items. He also compiled a questionnaire to measure adolescents’ relationship with friends, how comfortable they are with their friends, how big their circle of friends is and to what extent adolescents prefer to be with their friends. The questionnaire consists of 25 items. Both questionnaires were answered on a six-point scale ranging from ‘It is exactly how I experience it’ (6) to ‘It is definitely not how I experience it’ (1). The higher the obtained score, the better the relationship. The Cronbach’s Alpha Reliability Coefficient for the section on parent-adolescent relationship was 0.95 and 0.77 for the section on the relationship with friends.

### Procedure

The researchers visited all the respondents at their respective schools. Arrangements had to be made to ensure that the visits did not interfere with the academic programme of the school. Other factors, which had to be kept in mind, were fixed transport arrangements for the children, the daily routine of the parents, extra-curricular activities of the children and the house rules of the boarding school. The questionnaires were completed in the afternoon after the official school hours. At four of the schools, it was possible to administer the questionnaires to the respondents as a group, but at the remaining eight schools, individual sessions with respondents were necessary.

Most respondents were able to complete the questionnaire in 35 min but no time restriction for completing the questionnaires was set. Verbal instructions were given to the respondents for the completion of the questionnaire. The exact same instructions were also provided on the questionnaire. One of the researchers was constantly present during the completion of the questionnaires to clarify any language uncertainties. Four learners who spoke an African language at home voluntarily completed the developed questionnaire to identify possible language difficulties and uncertainties with regard to certain items in the questionnaire. Any ambiguities that arose were explained to the respondents. The respondents were asked to indicate their answers on the questionnaire. In some cases, the researcher, teacher, occupational therapists and physiotherapists assisted the respondents who experienced difficulties in fine motor skills which impacted on their writing. The questionnaires were carefully checked to ensure that they were fully completed before the data was captured.

## Results of the investigation

The first aim of the investigation was to establish whether certain factors facilitate or inhibit sports participation of adolescents with a mobility impairment. For this purpose, two groups were created: learners who participate in sport and those who do not. The two groups were then compared with regard to the variables in the three categories (the *inevitable, structure* and *personal* factors). In some instances, the variables provided discrete data (e.g. boys/girls or mainstream/special school). In such instances a *x*^2^-value was calculated, which appears in Table 1. Other variables such as relationships with parents and friends provided continuous data, which enabled the calculation of a *t*-value to test for significant differences between the means of learners who participate in sport and those who do not. The results appear in Table 2.

**TABLE 1:** Differences with regard to gender, type of school, hostel dwelling, time of acquired impairment and type of mobility impairment of learners who participate in sport and those who do not participate.

Categories	Sport participation	No participation	Total	*x^2^*- value
**Gender**				
Boys	58	20	78	*x^2^* (1) = 8.85 *p* = 0.0029
Girls	31	31	62	
Total	89	51	140	
**Type of school**				
Mainstream	7	1	8	*x^2^* (1) = 2.10 *p* = 0.1475
Special school	82	50	132	
Total	5	51	140	
**Stay in hostel**				
Yes	49	32	81	*x^2^* (1) = 0.79 *p* = 0.3753
No	40	19	59	
Total	89	51	140	
**Onset**				
Congenital	67	34	101	*x^2^* (1) = 1.20 *p* = 0.2739
Acquired	22	17	51	
Total	89	51	140	
**Mobility impairment**				
Spinal cord injury	24	19	43	*x^2^* (3) = 2.28 *p* = 0.5167
Cerebral palsy	31	13	44	
Spina bifida and bone deformity	17	8	25	
Muscular dystrophy	14	7	21	
Total	86	47	133	

**TABLE 2:** Differences with regard to age, health, social relationships and emotions of learners who participate in sport and those who do not participate.

Variable	Sport participation	*N*	Mean	*S*	*t*-value
Age	Yes	89	16.35	2.05	*t*(138) = 3.52
	No	51	17.63	2.10	*p* = 0.0006
Health	Yes	89	4.99	1.09	*t*(138) = 2.52
	No	51	4.43	1.51	*p* = 0.0130
Relationship with parents	Yes	89	205.00	38.98	*t*(138) = 0.14
	No	51	205.90	34.09	*p* = 0.8852
Relationship with friends	Yes	89	84.20	15.28	*t*(138) = 1.00
	No	51	81.45	16.21	*p* = 0.3177
Timid	Yes	89	16.78	4.29	*t*(138) = 1.33
	No	51	15.57	6.43	*p* = 0.1859
Trust	Yes	89	19.34	5.53	*t*(138) = 2.96
	No	51	16.31	6.27	*p* = 0.0036
Control	Yes	89	19.83	4.26	*t*(138) = 0.43
	No	51	20.22	6.25	*p* = 0.6668
Gregariousness	Yes	89	14.53	4.21	*t*(138) = 2.07
	No	51	12.84	5.29	*p* = 0.0400
Aggression	Yes	89	7.48	6.47	*t*(138) = 1.91
	No	51	10.04	9.34	*p* = 0.0586
Distrust	Yes	89	8.73	4.17	*t*(138) = 3.44
	No	51	11.27	4.28	*p* = 0.0008
Discontrol	Yes	89	11.08	4.55	*t*(138) = 0.38
	No	51	10.78	4.11	*p* = 0.7035
Depression	Yes	89	5.76	4.59	*t*(138) = 0.66
	No	51	6.31	4.92	*p* = 0.5077

From the results in Table 1, gender showed a significant difference between the two groups. More boys participated in sport compared to girls. In total 58 of the 78 boys (74%) with a mobility impairment participated in sport compared to girls where 31 of the 62 (50%) participated. Most of the boys in the sample participated in basketball and bowling followed by cycling. Most of the girls participated in basketball followed by soccer and cycling. Basketball and cycling seem to be popular sport types among boys and girls with a mobility impairment.

No significant differences were obtained with regard to the type of school the learners attended. Of the 8 learners in the sample who attended mainstream schools, 7 participated in sports (87%) while 82 of the 132 learners (62%) in special schools participated in sports. The observed difference was insignificant, probably because of the small number of learners in mainstream schools. Likewise, no significant differences were obtained with regard to hostel dwelling, the type of mobility impairment or the onset of the disability.

With regard to the results in Table 2, age and health showed significant differences. Learners with a mobility impairment who participate in sport were significantly younger compared to those who do not participate. As expected, those who participate in sport reported significantly better health conditions compared to the group that does not participate. No significant differences between the two groups were obtained regarding the support they enjoy from social relationships (relationship with parents and friends). With regard to the measured emotions, trust (and its opposite, distrust) showed a significant difference. Those learners with a mobility impairment who participate in sport have a significantly higher level of trust and a significantly lower level of distrust compared to those who do not participate. Learners who participate in sport also showed higher levels of gregariousness. No significant differences between the two groups could be shown with regard to timid, control, discontrol, aggression or depression.

The second aim was to identify the most important factors that differentiate best between the group that participates in sport and the group that does not participate in sport. For this purpose, a stepwise forward discriminant analysis was performed. The following independent variables were used: gender, type of school, hostel dwelling, onset of the impairment, type of impairment, age, health, relationship with parents and friends as well as the emotional constructs. The results appear in Table 3.

**TABLE 3:** Discriminant analysis of learners with a mobility impairment who participate in sport and those who do not participate.

Step	Variable	*R*^2^	*F*	*df*	*p*
1	Age	0.09	13.42	(1,131)	*p* = 0.0004
2	Trust	0.17	13.70	(2,130)	*p* = 0.0001
3	Gender	0.20	11.20	(3,129)	*p* = 0.0001
4	Health	0.22	9.49	(4,128)	*p* = 0.0001

In a discriminant analysis the independent variable that differs the most between the identified groups, enters the model first. In this instance, it was age that explained 9% of the variance in the distinctive characteristics of the group that participates in sport and the non-participating group. The proportion explained was significant: *F*(1,131) = 13.42; *p* < 0.01. The next variable to enter was trust explaining an additional 8% of the variance between the members of the two groups. This additional proportion was significant: *F*(2,130) = 13.70; *p* < 0.01. The third variable to enter the model was gender explaining 2% more of the differentiation not explained by age and trust: *F*(3,129) = 11.20; *p* < 0.01. Health was the last variable to enter the model. It explained an additional 2% of the distinctive characteristics of the two groups with *F*(4,128) = 9.49; *p* < 0.01. None of the remaining independent variables could explain a significant larger proportion of the variance between members of the two identified groups. In total, age, trust, gender and health explained 22% of the variance in the distinctive characteristics of the group that participates in sport and the non-participating group.

## Discussion of the results

From the results of the current investigation, four factors emerged which, to some extent, explain the difference between a group of adolescent learners with a mobility impairment who participate in sport and a group that does not participate. Three of the four factors fall in the *inevitable* category. No factor in the *structure* category (such as the type of school or hostel dwelling) made a significant contribution to the explained variance. Only one factor in the *personal* category, namely trust, explained a significant proportion of the variance between the learners in the two groups.

According to the results, age seems to be the most prominent differentiating factor, explaining 9% of the variance between the two identified groups. It seems that younger learners with a mobility impairment are more inclined to participate in sport than older learners. A possible reason might be that younger learners are less affected by negative comments and criticism on their sport participation compared to older learners. One of the manifestations of adolescent egocentrism is generally known as *imaginary audience,* which is an exaggeration of the attention adolescents believe other people are paying to them. They become preoccupied with how others may judge or evaluate them (Lin 2016:393). This behavioural pattern increases with age (Bester 2013:407; Frankenberger 2000:343–354), which might explain the reluctance of older learners to participate in certain activities, including sport-related activities. Although age has been identified as an important variable that contributes to the difference between learners who participate in sport and those who do not participate, the age when the disability occurred did not play any significant role. The onset of an individual’s mobility impairment causes great changes in all aspects of the individual’s life. Individuals with an acquired physical disability experience loss of independence that they previously enjoyed; loss of body integrity and mobility; loss of pre-existent roles and often a loss of social relationships (Psarra & Kleftaras 2013:79–99). Therefore, it is generally assumed that individuals with a congenital disability are in a better position to adapt to their circumstances (Bogart 2015:107–109). Based on this assumption it was expected that a difference in sport participation would exist between those learners who differ with regard to the onset of their disability. Such a difference could, however, not be shown in the current investigation.

Trust explained an additional 8% of the variance over and above the 9% already explained by age. According to the measuring instrument that was used, the trait ‘trust’ is an indication of emotional stability. It refers to the ability to control your emotions and to respond to crises in a calm and responsible way. People who possess trust accept the course of their lives, demonstrate hope and show confidence in themselves and other people. Trust is fundamental in any interpersonal relationship (Rotenberg et al. 2010:1086). Adolescents may not take a conscious decision to trust people, but they develop a sense of trust over time. The ability to trust other people is just as important as the feeling of being trusted by others (Preble 2015:433). Having opportunities to trust and to be trusted is, therefore, a crucial part of adolescents’ affective experiences and supports their capacity to enjoy a meaningful life (Rooney 2010:347). This is also applicable to a sports context and might possibly explain the higher level of trust (Table 2) for those who participate in sport, compared to those who do not participate.

Gender differences were also identified. More boys with a mobility impairment participated in sport compared to girls with a mobility impairment. This phenomenon also occurs in learners without a mobility impairment. According to Vilhjalmsson and Kristjansdottir (2003:370), studies have repeatedly shown that boys outnumber girls in team sports such as soccer and basketball, whereas girls outnumber boys in individual and medium to low-intensity sports such as gymnastics and swimming. More girls than boys engage in physical activity for appearance, health and fitness-related reasons and more boys for the sake of competition, demonstration of masculine ability, and the pursuit of victory (Vilhjalmsson & Kristjansdottir 2003:370). Slater and Tiggemann (2010:619–626) attempted to gain a deeper understanding of the reasons why girls ceased participation in sport and other physical activities. The girls in their sample provided a number of reasons, *inter alia* a loss of interest in the activity; sport interfering with social activities; competence; non-availability of sport at the school; injury; practical reasons such as transport arrangements; poor team relationships; conflict with other boys and girls; the coach and the influence of friends and family.

One has to accept that health will inhibit the sport participation of adolescents with a mobility impairment and, therefore, it is not surprising that health was identified as a variable that distinguishes between participating and non-participating learners. However, it should be mentioned that health was not identified as the most important variable. Health only explained an additional 2% of the variance that was not explained by age, trust and gender. Having a mobility impairment does not always imply that adolescents experience additional health issues. While some types of mobility impairment result in extensive healthcare needs, other conditions do not. Young et al. (2007:663–664) investigated health-related quality of life among children with cerebral palsy. Some of the children experienced pain, discomfort and tiredness but some of these children were proud of their accomplishments despite the problems that accompany their disability. According to Kostanjsek et al. (2011:1475–1482), adolescents may suffer from a variety of health conditions, but to obtain a full understanding of how they experience their health condition requires comprehensive information on the impact of the condition. Krahn, Walker and Correa-De-Araujo (2015:S198–S206) found that the impact of a mobility impairment varies according to the type of limitation and the condition underlying the impairment. Some adolescents who acquire a mobility impairment through injury can more readily differentiate their disability from their health status. For other adolescents, their health status may directly lead to their disability (e.g. diabetes that can lead to the loss of a limb). Many factors (e.g. socio-economic status, age, gender and quality of education) may affect the impact of the mobility impairment and may lead to even poorer health conditions.

## Conclusion

Three of the four factors that contributed to the distinctive characteristics of participating and non-participating learners fell within the *inevitable* category. These factors cannot directly be manipulated but there are guidelines that can be considered when dealing with these factors. Children with mobility impairment should be encouraged to start participating in sport at an early age. Younger children are less sensitive about the mistakes they make or comments made by other people. Older adolescents are more self-conscious, which may create resistance towards sport participation. The development of trust was the only manipulative variable which emerged as an important variable. This directs an appeal to parents, teachers and also sports coaches to support and encourage learners with a mobility impairment to foster trust in their lives. With more trust the possibility of taking responsible risks increases, which can facilitate their willingness to participate in sport. Since girls are less inclined to participate in sport, specific attention should be given to address their reluctance in this regard. Health is a factor that can inhibit sport participation; however, it should not be over emphasised. The emphasis should rather be on information regarding adaptive sport opportunities and encouragement to participate in an adaptive sport environment.

The investigation has limitations that open new possibilities for future research. In the current investigation, no significant difference in sport participation could be obtained between mobility-impaired learners in mainstream schools and those in special schools. However, only eight learners in the current sample attended mainstream schools, which complicates a final conclusion in this regard. The sport participation of mobility-impaired learners in a mainstream school needs to be investigated in more detail. It was mentioned earlier that information regarding adaptive sport could enhance the participation of mobility-impaired learners. Such information did not form part of the current investigation, which should be considered as a limitation. Information on adaptive sport has to be incorporated in future research as a possible factor that may explain why some learners participate in sport and others not. Only 22% of the variance between the two identified groups (participating and non-participating learners) could be explained with the independent variables used in the research project. That leaves 78% unexplained. There must be other variables that contribute to the proportion of the unexplained variance, which creates possibilities for future research. Not only information on adaptive sport but also variables such as interest, attitude, self-confidence and motivation can be considered in this regard.
